# The prevalence of antibiotic-resistant and multidrug-resistant bacteria in urine cultures from inpatients with spinal cord injuries and disorders: an 8-year, single-center study

**DOI:** 10.1186/s12879-022-07235-3

**Published:** 2022-03-09

**Authors:** Vladimír Šámal, Vít Paldus, Daniela Fáčková, Jan Mečl, Jaroslav Šrám

**Affiliations:** 1 Department of Urology, Krajská Nemocnice Liberec, Husova 10, 46063 Liberec, Czech Republic; 2 Department of Microbiology, Krajská Nemocnice Liberec, Liberec, Czech Republic; 3 Traumatology and Orthopedics Center, Krajská Nemocnice Liberec, Liberec, Czech Republic

**Keywords:** Multidrug resistance, Urinary tract infection, ESBL resistance, Spinal cord injury, Neurogenic bladder, Neurogenic lower urinary tract dysfunction

## Abstract

**Background:**

Patients, especially inpatients, with spinal cord lesions and disorders (SCI/D) have an elevated risk of recurrent urinary tract infections with multidrug resistant (MDR) bacteria. This study evaluated antimicrobial resistance and the prevalence of multidrug resistance and determined the risk factors for multidrug resistance.

**Methods:**

In this retrospective cohort study, urine culture results were used to calculate the antimicrobial resistance rate and the incidence of infection with MDR bacteria in the SCI/D population. MDR was defined as acquired nonsusceptibility to at least one agent from three or more antimicrobial categories. The cohort included 402 inpatients from 2013 to 2020, with 1385 urine isolates. We included only the first isolate; duplicate isolates, defined as positive cultures of the same strain within 14 days, were excluded from the evaluation.

**Results:**

The most common MDR strains were *Klebsiella* spp*.* (29%) and *Escherichia coli* (24%). MDR isolates were detected in 50% of the samples and extended spectrum beta-lactamase (ESBL)-producing isolates were detected in 26%, while carbapenem resistance was found in 0.1%. Significantly higher rates of infection with MDR bacteria were identified in groups of patients with indwelling urethral/suprapubic catheters (p = 0.003) and severity scores of C1–C4/AIS A–C (p = 0.01). We identified age (OR: 0.99, 95% CI; 0.98–0.99, p = 0.000), sex (OR: 1.55, 95% CI; 1.16–2.06, p = 0.003), management with urethral/suprapubic catheters (OR: 2.76, 95% CI; 2.04–3.74, p = 0.000), and spontaneous voiding (OR: 1.84, 95% CI; 1.03–3.29, p = 0.038) as independent predictors of multidrug resistance in our study population.

**Conclusions:**

We identified a high antibiotic resistance rate and an increasing prevalence of infection with MDR bacteria in the SCI/D inpatient population. Particular attention should be given to bladder management, with an emphasis on minimizing the use of indwelling catheters.

## Background

Urinary tract infections (UTIs) are very common in patients with spinal cord injuries and disorders (SCI/D). Positive urine culture was reported in 50–75% of these patients [1]. In general, each SCI/D patient had 2.5 UTIs per year [2]. UTIs are one of the most common complications of long-term treatment in SCI/D patients [3].

Increased bacterial resistance, especially multidrug resistance to antimicrobial agents, is now a major public health issue worldwide. Resistance to third-generation cephalosporins in *Escherichia coli* and *Klebsiella pneumoniae* is growing rapidly, primarily due to their production of extended-spectrum beta-lactamases (ESBLs), which are often associated with resistance to other antibiotics. Patients with multidrug resistant (MDR) *E. coli* and *K. pneumoniae* are often treated with carbapenems, but the number of carbapenem-resistant *Enterobacteriaceae* (CRE) isolates is increasing. In 2018, more than half of *E. coli* isolates and more than one-third of *K. pneumoniae* isolates were resistant to at least one antimicrobial group, and combined resistance was also common [4]. The increase in vancomycin-resistant isolates of *Enterococcus faecium* (VRE) is also a problem [4].

SCI/D patients repeatedly require health care and have a higher level of exposure to antibiotics, which increases the risk of infection with and colonization by MDR strains, especially by gram-negative bacteria (GNB). Infection with MDR strains is associated with a much worse outcome, prolonged length of stay, increased morbidity and mortality, and greater risk of impaired kidney function and urolithiasis, especially infectious urolithiasis [5–8]. Thus, the increase in infections with MDR bacteria is rapidly becoming a problem, especially in the inpatient setting.

In routine clinical practice, antimicrobial therapy is generally deployed upon the development of clinical signs of a UTI. To employ empirical treatments, which can be adjusted and targeted after receiving the results of antimicrobial susceptibility testing (AST), it is necessary to understand the current epidemiological context of uropathogens and the resistance rate to antimicrobial agents.

Our study was performed to assess resistance to the tested antimicrobial agents and the prevalence of infection with MDR strains in the SCI/D population with neurogenic lower urinary tract dysfunction (NLUTD) and to identify the risk factors for infection with MDR strains.

## Methods

### Study population

This was a retrospective cohort study focused on antimicrobial drug resistance and infection with MDR bacteria in the inpatient SCI/D population. We included patients hospitalized for SCI/D in the spinal care ward from 1 January 2013 to 31 December 2020 and obtained data from electronic medical records (EMRs) and the central database of the microbiology department. The main inclusion criterion was the development of NLUTD after SCI/D. There were no exclusion criteria. A total of 402 adult patients were enrolled, and six patients were excluded from the evaluation (four did not have SCI/D, two had incomplete data). A total of 396 patients were evaluated (303 men and 93 women) and 1101 urine samples were collected, from which 1385 bacterial isolates were obtained. From each patient, a mean of 2.5 urine samples were collected (one sample each from 140 patients; two samples each from 103 patients; three samples each from 64 patients, and ≥ 4 urine samples each from 89 patients). At the time of their first UTI, 55% of the patients had an indwelling urethral catheter (UC), 9% had a suprapubic catheter (SC), 23% had clean intermittent catheterization (CIC), and 13% of patients were managed with spontaneous voiding (SV). The detailed characteristics are given in Table 1.

**Table 1:** Demographic and background data of study population

Patient characteristic		Trauma	Non-trauma	Total	
		*N *(row %)	*N *(row %)	*N *	col %
		319 (80.6)	77 (19.4)	396	–
Age	Average (SD)	46 (16)	66 (12)	49 (17)	–
	median	45	67	50	–
	(min. max)	(15. 92)	(24. 89)	(15, 92)	–
Sex	Male	259 (85.5)	44 (14.5)	303	77
	Female	60 (64.5)	33 (35.5)	93	23
NLI^a^	C1–4	58 (84.1)	11 (15.9)	69	17
	C5–8	76 (88.4)	10 (11.6)	86	22
	T	128 (72.3)	49 (27.7)	177	44
	L,S	59 (88.1)	8 (11.9)	67	17
AIS^a^	A	101 (91.8)	9 (8.2)	110	28
	B	54 (80.6)	13 (19.4)	67	17
	C	71 (74.7)	24 (25.3)	95	24
	D	91 (74.0)	32 (26.0)	123	31
	E	4 (100.0)	0 (0.0)	4	1
Time since injury	< 1 year	231 (77.0)	69 (23.0)	300	70
	1—5 years	43 (91.5)	4 (8.5)	47	11
	6–10 years	19 (90.5)	2 (9.5)	21	5
	> 10 years	55 (93.2)	4 (6.8)	59	14
Bladder manag. ^a^	UC or SC	220 (76.9)	66 (23.1)	286	72
	CIC	115 (93.5)	8 (6.5)	123	31
	SV	31 (77.5)	9 (22.5)	40	10

^a^The total number of patients is not 396 as some patients may appear in more than one category

*NLI* neurogenic level of injury, *AIS* The American Spinal Injury Association Impairment scale, *UC* indwelling urethral catheter, *SC* suprapubic catheter, *CIC* clean intermittent catheterization, *SV* spontaneous voiding

### Urine specimen collection

In patients with spontaneous voiding, we used 5 ml of clean-catch midstream urine; in patients on the CIC regimen, urine was collected from the catheter. In patients managed with UC/SC, we used urine collected after catheter replacement. Urine collection was performed when clinical symptoms of UTI were observed, if UTI was suspected or for routine purposes.

### Urine culture

The collected urine samples were inoculated on chromogenic agar UriSelect4^®^ (Bio–Rad, France) within two hours. Samples taken outside working hours were stored at 2–8 °C according to preanalytical standards. An evaluation was performed after 18–24 h of aerobic incubation at 35 ± 2 °C. Bacterial isolates were identified according to colony morphology, Gram staining, and MALDI TOF MS^®^ mass spectrometry (Bruker, Daltonics, Germany). We considered a sample with a growth of ≥ 10^3^ colony-forming units/mL of primary pathogens to be positive.

### Antimicrobial susceptibility testing

The antibiotic disk diffusion method was used in accordance with the Guidelines and breakpoints of the European Committee on Antimicrobial Susceptibility Testing (EUCAST) [9]. AST was performed on Mueller–Hinton agar (Bio–Rad, France) using antibiotic disks (Bio–Rad, France). The evaluation was performed after 16–20 h of incubation at 36 °C. The measured inhibition zones were categorized according to the EUCAST guidelines as susceptible, intermediate (changed to “susceptible—increased exposure” in 2019), or resistant [9].

Only the first bacterial isolate per patient was included in the protocol; duplicate isolates were defined as positive cultures of the same isolate obtained within 14 days of the initial isolate. Different isolates were considered different individual isolates. Polymicrobial isolates were excluded if the individual components could not be identified.

In accordance with the European Center for Disease Prevention and Control, MDR bacteria were defined as those with acquired nonsusceptibility to at least one agent in three or more antimicrobial categories. Extensively drug-resistant (XDR) bacteria were defined as those with nonsusceptibility to at least one agent in all but two or fewer antimicrobial categories (i.e., the bacterial isolates remained susceptible to only one or two antimicrobial categories). Pandrug-resistant (PDR) bacteria were defined as those with nonsusceptibility to all agents in all antimicrobial categories [10].

*Enterobacterales* strains resistant to amoxicillin-clavulanic acid, and/or piperacillin-tazobactam and/or cefotaxime and/or meropenem were further tested using the AmpC & ESβL Detection Discs^®^ kit (MAST, France) and it was determined whether they were ESBL-producing strains. Strains of *Enterobacterales*, *Pseudomonas aeruginosa* and *Acinetobacter* spp*.* resistant to meropenem in patients that were positive for carbapenemase-producing *Enterobacterales* strains (according to a β Carba Test^®^; Bio–Rad, France) were assessed for carbapenemase production in the National Reference Laboratory for Antibiotics (SZÚ, Prague, CZ) by MALDI TOF and PCR. *Staphylococcus aureus* strains resistant to cefoxitin and oxacillin were considered strains of methicillin-resistant *S. aureus* (MRSA). Strains of *E. faecalis* and *E. faecium* resistant to vancomycin were classified as vancomycin-resistant.

### Study endpoints

- The primary objectives of this study were to evaluate resistance (and multidrug resistance) to the tested antibiotics in this population and determine risk factors for the development of multidrug resistance.
- The secondary objectives were to evaluate the prevalence of ESBL, carbapenem, and vancomycin resistance, as well as the prevalence of methicillin-resistant *Staphylococcus aureus*, extensive drug resistance and pandrug resistance.

### Statistical methods

We used the mean and standard deviation or the median and quartile values to describe continuous variables. To determine if the same number of patients were infected with MDR and non-MDR bacteria within each category (stratified by the variables sex, etiology, bladder management method, severity of injury, time since injury, and urinary culture), we used the Chi-square goodness-of-fit test. Univariate and multivariate logistic regression were used to determine independent predictors of multidrug resistance. The results are presented as adjusted odds ratios (ORs) with 95% confidence intervals (CIs). A significance level of 5% was used for all statistical tests. We used SPSS version 18 statistical software (IBM, IL, USA) for the statistical analysis. When the term significance is used in the text below, it means statistical significance.

## Results

We examined the results of the urine culture and AST of 1385 bacterial isolates. Gram-negative bacteria were the most common (1191, 86.8%); the rest were gram-positive cocci. The most common strains were *Klebsiella* spp*.* (402, 29%), *E. coli* (329, 24%), *P. aeruginosa* (180, 13%), *E. faecalis* (174, 12%), and *P. mirabilis* (133, 10%); other strains were much less common (Table 2).

**Table 2:** Overview of bacterial strains and MDR strains

Bacterial strain	N (%)	MDR N (%)	XDR N (%)	PDR N (%)	ESBL N (%)	CPE N (%)	MRSA N (%)
*Klebsiella species*	402 (29)	346 (86)	258 (64)	15 (4)	255 (63)	0 (0)	NA
*Escherichia coli*	329 (24)	98 (30)	32 (10)	0 (0)	43 (13)	0 (0)	NA
*Pseudomonas aeruginosa*	180 (13)	48 (27)	35 (19)	3 (2)	0 (0)	2 (1)	NA
*Enterococcus faecalis*	174 (13)	0 (0)	0 (0)	0 (0)	0 (0)	0 (0)	NA
*Proteus mirabilis*	133 (10)	61 (46)	3 (2)	0 (0)	14 (11)	0 (0)	NA
*Providencia stuartii*	51 (4)	51 (100)	21 (41)	0 (0)	28 (55)	0 (0)	NA
*Enterobacter species*	35 (3)	30 (86)	7 (20)	0 (0)	6 (17)	0 (0)	NA
*Morganella morganii*	27 (2)	27 (100)	8 (30)	0 (0)	2 (7)	0 (0)	NA
*Serratia marcescens*	19 (1)	17 (89)	0 (0)	0 (0)	3 (16)	0 (0)	NA
*Citrobacter koseri*	8 (1)	0 (0)	0 (0)	0 (0)	0 (0)	0 (0)	NA
*Enterobacter aerogenes*	7 (1)	6 (86)	2 (29)	0 (0)	2 (29)	0 (0)	NA
*Proteus vulgaris*	6 (0)	6 (100)	1 (17)	0 (0)	1 (17)	0 (0)	NA
*Enterococcus faecium*	5 (0)	0 (0)	0 (0)	0 (0)	0 (0)	0 (0)	NA
*Staphylococcus aureus*	4 (0)	0 (0)	2 (50)	0 (0)	0 (0)	0 (0)	3 (75)
*Acinetobacter baumannii c*	3 (0)	1 (33)	1 (33)	0 (0)	0 (0)	0 (0)	NA
*Citrobacter freundii*	2 (0)	1 (50)	1 (50)	0 (0)	0 (0)	0 (0)	NA
Total	1385 (100)	692 (50)	371 (27)	18 (1)	354 (26)	2 (0)	3 (0)

The overall prevalence of multidrug resistance in this cohort was 50% (Table 2). *P. stuarti*, *M. morganii*, and *P. vulgaris* were 100% MDR. In total, 27% of the strains were XDR, most of which were *Klebsiella* spp*.* (258, 64%), *P. stuarti* (21, 41%), and *P. aeruginosa* (35, 19%). Only 1% of strains were PDR. ESBL resistance was found in 26% of strains. The most common producers of ESBL were *Klebsiella* spp*.* (255, 63%) and *P. stuarti* (28, 55%). CPE resistance was identified in only 2 strains of *P. aeruginosa*. We did not observe MRSA or VRE strains.

**Fig. 1:**
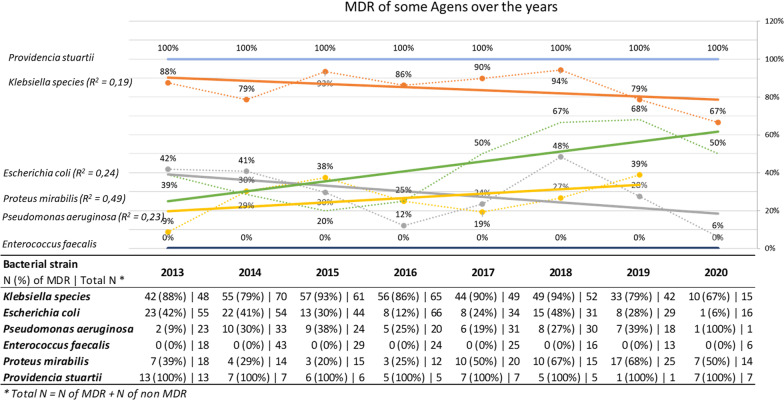
Proportion of main MDR strains by study year. Figure 1 is a line graph of the proportion of the main MDR strains by study year. Linear trends, determined by using the coefficient of determination (R^2^), demonstrate that there is no significant linear increase/decrease in the prevalence of MDR strains. The overall prevalence of other MDR strains is demonstrated in Table 2

The proportions of MDR strains over the study period are shown in Fig. 1. MDR strains of *P. vulgaris* became increasingly common, while the MDR strains of other species remained stable. As shown in Table 3, we found that MDR strains were significantly more common in the group of patients managed with UC/SC (p = 0.003). MDR strains were identified significantly less often in women (p = 0.006) and in patients managed with CIC (p = 0.000).

**Table 3:** Prevalences of MDR and non-MDR strains in groups of patients

	MDR N (%)	Non MDR (N %)	p-value
Sex			
Male	579 (52)	535 (48)	0.187
Female	113 (42)	158 (58)	**0.006**
Etiology			
Non-traumatic	104 (47)	118 (53)	0.347
Traumatic	588 (51)	575 (49)	0.703
Bladder management			
UC or SC	559 (55)	464 (45)	**0.003**
CIC	105 (36)	186 (64)	**0.000**
SV	28 (39)	43 (61)	0.075
Severity of injury			
C1–C4, AIS A or B or C	133 (59)	94 (41)	**0.010**
C5–C8, AIS A or B or C	120 (47)	134 (53)	0.380
T1–S5, AIS A or B or C	293 (50)	290 (50)	0.901
AIS D	145 (46)	171 (54)	0.144
Time since injury			
< 1 year	467 (49.0)	486 (51.0)	0.538
1–5 years	78 (56.5)	60 (43.5)	0.125
6–10 years	37 (50.0)	37 (50.0)	1.000
> 10 years	110 (50.0)	110 (50.0)	1.000
Urinary culture			
Gram-negative	692 (58)	510 (42)	**0.000**
Gram-positive	0 (0)	183 (100)	**0.000**

Bold value means singificant values

*AIS* The American Spinal Injury Association Impairment scale, *UC* indwelling urethral catheter, *SC* suprapubic catheter, *CIC* clean intermittent catheterization, *SV* spontaneous voiding

An overview of the rates of isolate resistance to the tested antibiotics is shown in Table 4. The results are based on AST. The resistance rates of isolates accounting for < 1% of the total isolates are not listed in the table. The overall levels of resistance to aminopenicillins and amoxicillin-clavulanic acid were extremely high at 70% and 48%, respectively. The overall level of resistance to piperacillin-tazobactam was 35%. The overall level of resistance to cefuroxime was 52%, while the levels of resistance to the third- and fourth-generation cephalosporins (cefotaxime, ceftazidime and cefepime) were 43%, 22% and 20%, respectively. The overall level of resistance to ciprofloxacin was also high, at 49%. Among aminoglycosides (gentamicin and amikacin), the overall levels of resistance were 37% and 4%, respectively. We found a very low level of resistance to meropenem (4%). The level of resistance to sulfamethoxazole-trimethoprim was 63%. None of the enterococci were resistant to vancomycin.

**Table 4:** Antibiotic resistance rates of main isolates from urine culture

*Bacterial strain*	*Klebsiella species*^a^		*Escherichia coli*^a^		*Proteus mirabilis*^a^		*Providencia stuartii*^a^		*Enterobacter species*^a^		*Morganella morganii*^a^		*Serratia marcescens*^a^	
Antibiotics	Resistant N (%)	Total N	Resistant N (%)	Total N	Resistant N (%)	Total N	Resistant N (%)	Total N	Resistant N (%)	Total N	Resistant N (%)	Total N	Resistant N (%)	Total N
Amikacin	13 (3)	398	3 (1)	329	1 (1)	132	1 (2)	51	1 (3)	35	0 (0)	26	0 (0)	19
Aminopenicilin	402 (100)	402	200 (61)	329	81 (61)	132	51 (100)	51	35 (100)	35	27 (100)	27	19 (100)	19
Amoxicillin-CA^b^	292 (73)	402	53 (16)	329	7 (5)	133	51 (100)	51	34 (97)	35	24 (89)	27	19 (100)	19
Cefepim	–	–	–	–	–	–	–	–	–	–	–	–	–	–
Cefotaxim	304 (76)	402	53 (16)	329	15 (11)	132	27 (53)	51	14 (40)	35	11 (41)	27	7 (37)	19
Ceftazidim	–	–	–	–	–	–	–	–	–	–	–	–	–	–
Cefuroxim	310 (78)	395	57 (18)	315	18 (15)	121	41 (85)	48	30 (100)	30	23 (88)	26	17 (100)	17
Ciprofloxacin	288 (72)	401	96 (29)	329	63 (47)	133	47 (92)	51	6 (17)	35	12 (44)	27	1 (5)	19
Colistin	–	–	–	–	–	–	–	–	–	–	–	–	–	–
Gentamicin	259 (64)	402	39 (12)	329	52 (39)	132	24 (47)	51	6 (17)	35	11 (42)	26	0 (0)	19
Gentamicin^c^	–	–	–	–	–	–	–	–	–	–	–	–	–	–
Chloramphenicol	–	–	–	–	–	–	1 (100)	1	–	–	0 (0)	1	–	–
SMX-TMP^d^	337 (84)	401	145 (44)	328	88 (66)	133	37 (73)	51	6 (17)	35	14 (52)	27	1 (5)	19
Linezolid	–	–	–	–	–	–	–	–	–	–	–	–	–	–
Meropenem	1 (0)	372	0 (0)	290	0 (0)	120	0 (0)	42	0 (0)	33	0 (0)	25	0 (0)	17
Nitrofurantoin	–	–	–	–	–	–	–	–	–	–	–	–	–	–
Piperacilin–tazobactam	277 (70)	395	40 (12)	327	1 (1)	132	10 (20)	51	14 (41)	34	10 (37)	27	6 (32)	19
Tigecyklin	–	–	–	–	–	–	–	–	–	–	–	–	–	–
Vankomycin	–	–	–	–	–	–	–	–	–	–	–	–	–	–

*Bacterial strain*	*Pseudomonas aeruginosa*		*Enterococcus faecalis*		*Total of Agens*	
Antibiotics	Resistant N (%)	Total N	Resistant N (%)	Total N	Resistant N (%)	Total N
Amikacin	23 (18)	131	–	–	42 (4)	1121
Aminopenicilin	–	–	1 (1)	174	816 (70)	1169
Amoxicillin-CA^b^	–	–	–	–	480 (48)	996
Cefepim	36 (20)	180	–	–	36 (20)	180
Cefotaxim	–	–	–	–	431 (43)	995
Ceftazidim	39 (22)	180	–	–	39 (22)	180
Cefuroxim	–	–	–	–	496 (52)	952
Ciprofloxacin	65 (36)	179	–	–	578 (49)	1174
Colistin	0 (0)	20	–	–	0 (0)	20
Gentamicin	44 (25)	179	–	–	435 (37)	1173
Gentamicin^c^	–	–	74 (43)	173	74 (43)	173
Chloramphenicol	–	–	–	–	1 (50)	2
SMX-TMP^d^	–	–	–	–	628 (63)	994
Linezolid	–	–	0 (0)	60	0 (0)	60
Meropenem	41 (23)	179	–	–	42 (4)	1078
Nitrofurantoin	–	–	0 (0)	174	0 (0)	174
Piperacilin–tazobactam	51 (28)	180	–	–	409 (35)	1165
Tigecyklin	–	–	0 (0)	174	0 (0)	174
Vankomycin	–	–	0 (0)	172	0 (0)	172

^a^Enterobacterales

^b^CA clavulanic acid

^c^Gentamycin for synergy

^d^SMX-TMP- sulfomethoxazol-trimetoprim

Overall, the lowest levels of resistance (< 10%) were to meropenem and amikacin.

We identified specific risk factors associated with the isolation of MDR strains. Based on the univariate analysis, we identified four variables associated with the development of MDR strains, namely, age, sex, bladder management method and severity of the injury (Table 5). We used the enter method of multivariate logistic regression (Table 6) to identify the variables that were independently associated with the identification of MDR strains. Increased age (OR: 0.99, 95% CI; 0.98–0.99, p = 0.000), male sex (OR: 1.55, 95% CI; 1.16–2.06, p = 0.003), UC/SC bladder management (OR: 2.76, 95% CI; 2.0–3.74, p = 0.000), and SV bladder management (OR: 1.84, 95% CI: 1.03–3.29, p = 0.038) were found to be independent predictors of the development of MDR strains in our study population.

**Table 5:** Univariate analysis of the risk of multidrug resistance

Variable	MDR N (%)	Non MDR N (%)	Adjust OR	95% CI	p-value
Age (mean ± SD)	48 ± 17	51 ± 18	0.99	0.98–0.99	**0.002**
Sex					
Male	579 (52)	535 (48)	1.51	1.16–1.98	**0.003**
Female	113 (42)	158 (58)	Reference		
Etiology					
Non-traumatic	104 (47)	118 (53)	Reference		
Traumatic	588 (51)	575 (49)	1.16	0.87–1.55	0.311
Bladder management					
CIC	105 (36)	186 (64)	Reference		
UC/SC	559 (55)	464 (45)	2.13	1.63–2.79	**0.000**
SV	28 (39)	43 (61)	1.15	0.68–1.97	0.599
Severity of injury					
AIS D	145 (46)	171 (54)	Reference		
C1–C4, AIS A or B or C	133 (59)	94 (41)	1.67	1.18–2.36	**0.004**
C5–C8, AIS A or B or C	120 (47)	134 (53)	1.06	0.76–1.47	0.747
T1–S5, AIS A or B or C	293 (50)	290 (50)	1.19	0.91–1.57	0.211
Time since injury					
< 1 year	467 (49.0)	486 (51.0)	Reference		
1–5 years	78 (56.5)	60 (43.5)	1.35	0.94–1.94	0.100
6–10 years	37 (50.0)	37 (50.0)	1.04	0.65–1.67	0.869
> 10 years	110 (50.0)	110 (50.0)	1.04	0.78–1.40	0.790

Bold value means singificant values

*AIS* The American Spinal Injury Association Impairment scale, *UC* indwelling urethral catheter, *SC* suprapubic catheter, *CIC* clean intermittent catheterization, *SV* spontaneous voiding

**Table 6:** Multivariate analysis

Variable	Adjust OR	95% CI	p-value
Age	0.99	0.98–0.99	**0.000**
Sex			
Male	1.55	1.16–2.06	**0.003**
Female	Reference		
Etiology			
Non traumatic	1.09	0.78–1.53	0.615
Traumatic	Reference		
Bladder management			
CIC	Reference		
UC or SC	2.76	2.04–3.74	**0.000**
SV	1.84	1.03–3.29	**0.038**
Severity of injury			
AIS D	Reference		
C1–C4, AIS A or B or C	0.95	0.64–1.40	0.795
C5–C8, AIS A or B or C	0.71	0.50–1.02	0.067
T1–S5, AIS A or B or C	0.98	0.73–1.32	0.903
Time since injury			
< 1 year	Reference		
1–5 years	1.29	0.88–1.88	0.188
6–10 years	1.23	0.74–2.03	0.423
> 10 years	1.26	0.92–1.73	0.157
Constant	1.23		0.558

Bold value means singificant values

*AIS* The American Spinal Injury Association Impairment scale, *UC* indwelling urethral catheter, *SC* suprapubic catheter, *CIC* clean intermittent catheterization, *SV* spontaneous voiding

## Discussion

Our work provides an overview of urine culture results and AST in spinal ward patients over a period of eight years. We evaluated all positive urinary findings to obtain an overview of the rates of antibiotic resistance, enzymatically conditioned resistance, and multidrug resistance. Knowledge of these parameters is important for selecting a specific antimicrobial therapy before the final results of AST are obtained. Many infected SCI/D patients are in critical condition, and knowledge of the epidemiological data and estimated resistance rates in these patients can affect the success of empirical antibiotic therapy. Infection with MDR strains increases patients’ morbidity and mortality, increases the likelihood of rehospitalization, prolongs the length of stay, and has a significant effect on the cost of treatment [11].

Because the primary objective of this study was to determine the prevalence of uropathogens and the rates of resistance, including multidrug resistance, we did not consider whether these infections were symptomatic UTIs or asymptomatic bacteriuria. Indeed, a detailed assessment of this aspect would be error-prone given the retrospective nature of our study, which was based on data from EMRs.

We used the current definition of multidrug resistance, which was adopted based on the consensus reached by an international expert panel in 2011 [10]. These guidelines established epidemiologically significant categories of antibiotics for each group of bacteria and defined MDR bacteria as those with nonsusceptibility to at least one agent in ≥ 3 antimicrobial categories. Most of the studies conducted before this consensus used a different definition of MDR bacteria, which was usually less strict. Therefore, it is difficult to compare our results with those of studies published before 2011.

In our study, which exclusively involved inpatients, 50% of the isolates were MDR bacteria, and the proportion of XDR bacteria was relatively high (27%). Fitzpatrick et al. found that 36.1% of GNB isolated from urine were MDR strains, one-fifth of which were obtained from outpatients [12]. The most common uropathogens were *E. coli* (27%), *K. pneumoniae* (16%) and *P. aeruginosa* (17.3%). They observed a significant shift among the resistant pathogens from gram-positive cocci to GNB over 9 year of follow-ups. Significant geographical differences in MDR bacteria were also observed. The results of other studies on the SCI/D population have shown a prevalence of MDR at rates of 60.7%, 41.3%, or 33% [11, 13, 14]. In general, an increase in the prevalence of resistant strains in recent years has been reported in several other studies [12, 15, 16]. Large regional differences in the occurrence and proportion of MDR strains have also been reported [11, 14].

In our cohort, the most common strains were *Klebsiella* spp*.* (29%), *E. coli* (24%) and *P. aeruginosa* (13%). Most similar studies have reported that *E. coli* is the dominant uropathogen in SCI/D patients, with a significantly lower proportion of *Klebsiella* spp*.* [12, 14, 17]. The relatively high proportion of patients managed with UC/SC, due to the acute nature of the spinal ward, is a possible reason for the high incidence of infection with *Klebsiella* spp*.* in our group. Most patients in this ward are hospitalized for an average of three months after injury before being transferred to a special rehabilitation institution for patients with SCI/D. In the present cohort, 15% of patients with polytrauma were receiving long-term management with UC/SC. This may partially explain the high prevalence of MDR strains and the identification of nosocomial strains of *Klebsiella* spp*.* The frequent use of broad-spectrum antibiotics for indications other than UTIs, which leads to the selection of MDR strains, may also explain these findings.

We observed an increase in ESBL production in *Enterobacterales *(26%). Prior administration of fluoroquinolones and third- and fourth-generation cephalosporins appears to be a risk factor for ESBL production [18]. The increasing trend in ESBL production is supported by another study that identified ESBL production in 6.6% of *E. coli* and *K. pneumoniae* strains [19]. We did not observe carbapenem resistance in our cohort; the estimated rate of CRE in SCI/D patients in other studies was 1.7–7.6% [13, 20]. Compared with other studies, this study reported a lower prevalence of MRSA [6, 21]. Thus, there is a clear trend in recent years, with a shift among MDR bacteria from gram-positive cocci to GNB [6].

Based on our overview of bacterial strains and the rates of resistance to various antibiotics, there is clear evidence of a high proportion of nosocomial strains, mostly GNB. Bacterial colonization occurs through the spread of strains derived from the intestinal microflora, perineum, or urethra when the catheter is manipulated [22]. Contamination from the patient’s external environment and transmission between patients and by medical staff are also common. Colonization can persist in the long term without any signs of an acute UTI. However, if colonization occurs, the patient is at risk for lifelong recurrent UTIs. The prevalence of multidrug resistance and other types of resistance in the general population varies between hospitals, wards and specific patient populations. The prevalence of resistance is influenced by the specific patient population, antibiotic policies and established clinical practices.

Bacterial antimicrobial resistance is usually genetically encoded. In addition to the traditional method of antimicrobial susceptibility testing, sequencing-based methods expand our ability to assess antimicrobial resistance. Whole-genome sequencing to detect genetic determinants of antimicrobial resistance is available and provides rapid and sensitive determination of resistance [23].

In our work, UC/SC bladder management, male sex, and injury severity were identified as risk factors for multidrug resistance. Other studies have reported similar findings [19, 24–26]. The most common risk factor for MDR was management with UC/SC. Other risk factors include a history of UTIs, previous antimicrobial therapy, and prolonged and repeated exposure to antimicrobials [27]. One of the basic methods for preventing multidrug resistance should be the early removal of indwelling catheters, as the prophylactic effect of management with CIC has been shown [28–31]. Another risk factor for MDR was spontaneous voiding. We consider this to indicate long-term colonization that persisted after switching from UC/SC to spontaneous voiding management methods. Risk factors for acquired resistance in different species have also been identified, but a detailed assessment of these factors is beyond the scope of this study.

We found a high level of resistance to antibiotics, especially aminopenicillins, amoxicillin-clavulanic acid, cephalosporins, fluoroquinolones and SMX-TMP, which are commonly used to treat UTIs. Other studies have found similar results [12, 26, 32].

Our work is limited by a number of factors. First, it was a retrospective study with data collected from one center. Although the sample size was relatively large, the findings need to be validated in a multicenter study. Second, clinically symptomatic infections and asymptomatic bacteriuria were not considered separately. The colonization of the lower urinary tract by MDR bacteria in patients with neurogenic bladder is generally high. Thus, the difference between symptomatic UTI and asymptomatic bacteriuria merits further investigation. Future studies are needed. Third, the results of this study could have been affected by regional trends, established clinical practices, and local antibiotic policies.

## Conclusion

In this large cohort of SCI/D inpatients with neurogenic lower urinary tract dysfunction, we observed increasing resistance among uropathogens and a high prevalence of MDR strains. In particular, the use of an indwelling catheter is a risk factor for infection with MDR bacteria.

## Data Availability

The datasets generated and/or analyzed during the current study are not publicly available because the local ethics committee require oversight of use of research data but are available from the corresponding author on reasonable request.

## Abbreviations

AST: Antimicrobial susceptibility testing
CFU: Colony forming unit
CIC: Clean intermittent catheterization
CRE: Carbapenem resistance
ESBL: Extended-spectrum beta-lactamases
GNB: Gram-negative bacteria
MDR: Multidrug resistant
PDR: Pandrug resistant
SCI/D: Spinal cord injury and disorders
VRE: Vancomycin resistance
UTI: Urinary tract infection
XDR: Extensively drug resistant
